# Psychological Capital and Mental Health in Ecuadorian University Students: The Mediating Role of Negative Stress

**DOI:** 10.3390/ejihpe16060076

**Published:** 2026-05-28

**Authors:** Lucía Quinde, Victor López-Guerra, Sandra Guevara-Mora

**Affiliations:** Faculty of Behavioral Sciences, Universidad Técnica Particular de Loja, Loja 110107, Ecuador; dlquinde@utpl.edu.ec (L.Q.); srguevara@utpl.edu.ec (S.G.-M.)

**Keywords:** psychological capital, negative stress, anxiety and depression, psychological inflexibility, university students

## Abstract

This study examined the mediating role of negative stress in the relationship between Psychological Capital (PsyCap) and psychological distress indicators among Ecuadorian university students. PsyCap was conceptualized as a higher-order construct composed of hope, self-efficacy, resilience, and optimism. A cross-sectional study was conducted with 1732 university students (55% women; M = 20.44, SD = 2.29) from three Ecuadorian universities using validated self-report measures. Structural equation modeling supported the proposed mediational model and demonstrated an adequate fit to the data, χ^2^(367) = 1732, *p* < 0.001, CFI = 0.972, TLI = 0.969, RMSEA = 0.061 (90% CI [0.058, 0.063]), and SRMR = 0.041. PsyCap showed a significant negative association with negative stress (β = −0.311, *p* < 0.001). In turn, negative stress was positively associated with anxiety–depression symptoms (β = 0.785, *p* < 0.001) and psychological inflexibility (β = 0.774, *p* < 0.001). Mediation analyses revealed significant indirect effects of PsyCap on anxiety–depression (β = −0.244, *p* < 0.001) and psychological inflexibility (β = −0.241, *p* < 0.001) through negative stress. Direct effects remained significant but smaller in magnitude (β = −0.131 and β = −0.107, respectively), supporting a partial mediation model. The model explained 69.7% of the variance in anxiety–depression and 66.3% of the variance in psychological inflexibility. These findings suggest that PsyCap functions primarily as a protective psychological resource through its capacity to reduce maladaptive stress responses, which subsequently influence broader transdiagnostic indicators of psychological distress. The study highlights the relevance of integrating strengths-based approaches and stress-reduction strategies in university mental health interventions. Furthermore, it provides empirical evidence from a Latin American context, contributing to the understanding of mechanisms linking positive psychological resources and mental health among university students.

## 1. Introduction

The deterioration of mental health among young people has become a major global public health concern, particularly among university students, who represent one of the populations most vulnerable to psychological distress (Copeland et al., 2013; Gómez-Restrepo et al., 2025). In recent years, symptoms of anxiety and depression have increased considerably among adolescents and emerging adults, negatively affecting academic performance, emotional adjustment, interpersonal functioning, and overall well-being (Moreta-Herrera et al., 2021). This situation was further exacerbated by the COVID-19 pandemic, which intensified emotional distress worldwide and exposed the fragility of mental health systems in both developed and developing countries (Finch et al., 2025; Moreno-Montero et al., 2024).

University students are particularly vulnerable because they are exposed to multiple academic, social, and economic stressors, including academic overload, financial strain, sociocultural adjustment, performance pressure, and uncertainty regarding their professional future (Spence et al., 2022; Galve-González et al., 2024; Moreno-Montero et al., 2024). Persistent exposure to these demands may compromise emotional adjustment and increase susceptibility to internalizing symptoms and maladaptive coping responses (Pascoe et al., 2020).

Mental health is increasingly conceptualized as a continuum ranging from optimal psychological functioning to severe emotional dysfunction (Khan et al., 2024; Visser & Law-van Wyk, 2021). Consequently, contemporary psychological research has progressively shifted from deficit-oriented approaches focused exclusively on psychopathology toward perspectives emphasizing resilience, adaptive functioning, and protective psychological resources. Within this framework, positive psychology proposes that psychological strengths and personal resources play a central role in promoting well-being and protecting individuals against emotional maladjustment (Seligman, 2011).

This perspective is especially relevant in Latin American contexts, where socioeconomic inequality, economic instability, urban violence, and limited access to mental health services may intensify students’ vulnerability to maladaptive stress processes (Gómez-Restrepo et al., 2025). In Ecuador, these challenges may be even more pronounced due to structural inequalities and the limited availability of university psychological support systems. Despite the growing prevalence of emotional difficulties among Ecuadorian university students, empirical evidence examining protective psychological resources and transdiagnostic vulnerability mechanisms in this population remains scarce. Recent evidence from Ecuador identified perceived stress and psychological inflexibility as central transdiagnostic processes associated with anxiety and depression symptoms among university students (Vaca-Gallegos et al., 2025).

Among the psychological variables most consistently associated with emotional maladjustment in university populations is perceived stress. Perceived stress refers to the appraisal of life situations as unpredictable, uncontrollable, and overwhelming, exceeding the individual’s perceived coping resources (Remor, 2006; Ruisoto et al., 2020). According to Lazarus and Folkman’s (1984) transactional model of stress and coping, stress responses emerge not solely from external demands, but from individuals’ cognitive appraisal of those demands relative to available coping resources. From this perspective, psychological adjustment depends not only on environmental demands, but also on the individual’s capacity to interpret stressful situations as manageable and controllable. Consequently, stress responses are influenced not only by objective environmental demands, but also by the perceived availability of psychological resources that shape how stressful situations are cognitively interpreted and emotionally regulated (Lazarus & Folkman, 1984; Hobfoll, 2002; Luthans et al., 2007a).

Recent evidence suggests that not all stress responses are equivalent. Maladaptive stress experiences characterized by uncontrollability, emotional overload, hopelessness, and dysfunctional cognitive appraisal appear to be more strongly associated with psychological dysfunction and emotional symptoms (Ruisoto et al., 2022). In the present study, this maladaptive dimension is conceptualized as negative stress. Importantly, negative stress is not conceptualized as a synonym for anxiety, depressive affect, or general distress, but rather as a maladaptive appraisal subtype of perceived stress characterized by uncontrollability, emotional overload, hopelessness, and dysfunctional cognitive evaluation of environmental demands (Ruisoto et al., 2022; Moreno-Montero et al., 2024). This distinction is theoretically relevant because it conceptualizes negative stress as an antecedent cognitive–emotional appraisal process rather than as a direct emotional symptom. Recent transdiagnostic perspectives further suggest that maladaptive appraisal processes may function as shared vulnerability mechanisms contributing to emotional dysregulation and internalizing symptoms across multiple forms of psychological maladjustment (Dalgleish et al., 2020; McEvoy et al., 2010).

The conceptualization of negative stress as a maladaptive appraisal process is further supported by Seligman’s theory of learned optimism and explanatory style, which proposes that the cognitive interpretation of adverse events influences coping responses and psychological adjustment (Seligman, 1990). From this perspective, pessimistic appraisals are associated with helplessness, avoidance, and increased vulnerability to anxiety and depression, whereas optimistic explanatory styles facilitate adaptive coping and resilience. Contemporary evidence additionally suggests that stress-related cognitive appraisal processes are closely associated with emotional regulation capacities, coping flexibility, and vulnerability to internalizing symptoms among university populations (Finch et al., 2025; Moreno-Montero et al., 2024). Together, these perspectives support the notion that maladaptive stress appraisal may represent a shared underlying mechanism contributing to emotional maladjustment.

Another important transdiagnostic vulnerability factor associated with psychological maladjustment is psychological inflexibility. Psychological inflexibility refers to rigid patterns of experiential avoidance and cognitive fusion that interfere with adaptive functioning and value-consistent behavior (Bond et al., 2011; Ruiz et al., 2013). Individuals characterized by high psychological inflexibility tend to avoid unpleasant internal experiences even when such avoidance compromises long-term goals and psychological adjustment. Within transdiagnostic frameworks, maladaptive stress appraisal processes may intensify emotional dysregulation, experiential avoidance, and cognitive fusion, thereby increasing vulnerability to anxiety–depression symptoms and psychological inflexibility (Hayes et al., 2006; Kashdan & Rottenberg, 2010; Levin et al., 2014). Thus, the proposed model assumes that maladaptive stress appraisal processes may represent shared underlying mechanisms contributing to multiple forms of emotional maladjustment.

Within the framework of positive psychology, Psychological Capital (PsyCap) has emerged as one of the most relevant psychological resources for understanding adaptive functioning and mental health outcomes in academic contexts. PsyCap is conceptualized as a higher-order construct composed of self-efficacy, hope, resilience, and optimism (Luthans et al., 2004; López-Guerra et al., 2023). Self-efficacy refers to confidence in mobilizing cognitive and behavioral resources to cope with situational demands, whereas hope involves agency and pathway thinking directed toward goal attainment (Luthans et al., 2015). Optimism reflects positive expectations regarding future outcomes despite adversity (Seligman, 2002), and resilience represents the capacity to adapt successfully to stress and adversity while maintaining psychological functioning (Coutu, 2002; King et al., 2020). Unlike stable personality traits, these dimensions are considered state-like and therefore susceptible to development through psychoeducational and behavioral interventions (Luthans et al., 2007b; King et al., 2020). Collectively, these dimensions may function as proactive psychological resources capable of influencing how individuals perceive, interpret, and regulate stressful experiences (Luthans et al., 2007b; Avey et al., 2011; Youssef-Morgan & Luthans, 2015).

Previous research has consistently linked PsyCap with greater psychological well-being, academic engagement, resilience, and adaptive coping (Datu & Valdez, 2016; Siu et al., 2014). More recent evidence suggests that PsyCap may buffer the impact of stress-related maladjustment and emotional symptoms among university students (Finch et al., 2020; Moreno-Montero et al., 2024; Zewude et al., 2024). Collectively, these findings suggest that PsyCap functions as a protective psychological resource capable of reducing vulnerability to maladaptive stress responses. However, beyond functioning as a moderator or mediator, PsyCap may also operate as an antecedent psychological resource capable of shaping stress appraisal processes themselves (Lazarus & Folkman, 1984; Hobfoll, 2002; Avey et al., 2011). According to transactional stress theory, individuals with stronger psychological resources may be more likely to evaluate stressful situations as manageable and controllable rather than threatening or overwhelming.

Despite these advances, several conceptual and empirical gaps remain unresolved. First, previous studies have predominantly conceptualized PsyCap as a mediator or moderator of stress-related outcomes, whereas comparatively little attention has been given to its role as an antecedent psychological resource capable of shaping stress appraisal processes. Second, most investigations have relied on broad measures of perceived stress without differentiating maladaptive appraisal responses associated with uncontrollability, emotional overload, and hopelessness. Third, prior research has generally examined isolated indicators of maladjustment rather than integrating multiple transdiagnostic mechanisms within a unified explanatory framework. Finally, evidence from Latin American university populations remains scarce despite the increasing mental health burden observed in these contexts.

The present study addresses these gaps through an integrated framework grounded in positive psychology, transactional stress theory, and transdiagnostic perspectives. Specifically, this research conceptualizes Psychological Capital as a proactive psychological resource that may influence how individuals cognitively evaluate stressful experiences, negative stress as a maladaptive cognitive–emotional appraisal mechanism, and anxiety–depression symptoms together with psychological inflexibility as downstream manifestations of transdiagnostic emotional vulnerability processes.

To our knowledge, no previous study has simultaneously examined Psychological Capital as an antecedent psychological resource, negative stress as a maladaptive transdiagnostic appraisal mechanism, and both anxiety–depression symptoms and psychological inflexibility within a unified structural equation model in Latin American university students. Consequently, the present study addresses an important theoretical and empirical gap by proposing an integrated framework for understanding how protective psychological resources and maladaptive stress appraisal processes interact to influence mental health outcomes among university students.

Based on this framework, the following hypotheses were formulated:

**H1.**  *Psychological Capital will be negatively associated with negative stress because individuals with greater hope, optimism, resilience, and self-efficacy are expected to cognitively appraise stressful experiences as more manageable and controllable.*

**H2.**  *Negative stress will be positively associated with anxiety–depression symptoms and psychological inflexibility because maladaptive stress appraisal processes may intensify emotional dysregulation, experiential avoidance, and cognitive fusion.*

**H3.**  *Psychological Capital will show direct negative effects on anxiety–depression symptoms and psychological inflexibility due to its protective role in promoting adaptive emotional regulation, resilience, and coping resources.*

**H4.**  *Negative stress will mediate the relationship between Psychological Capital and both anxiety–depression symptoms and psychological inflexibility because cognitive stress appraisal processes may represent a central mechanism linking psychological resources with emotional maladjustment.*

**H5.**  *Indirect effects of Psychological Capital on psychological distress outcomes through negative stress will be stronger than direct effects, supporting a partial mediation model in which maladaptive stress appraisal operates as a key transdiagnostic mechanism underlying emotional vulnerability.*

## 2. Materials and Methods

### 2.1. Participants

The study sample consisted of 1732 undergraduate students enrolled in face-to-face programs at three universities in Loja, Ecuador: Universidad Técnica Particular de Loja (n = 792), Universidad Nacional de Loja (n = 753), and Universidad Internacional del Ecuador—Loja campus (n = 187). Participants were recruited through non-probability convenience sampling.

Inclusion criteria were: (a) being enrolled in the university for at least one academic year, (b) being 18 years of age or older, (c) having internet access, (d) possessing sufficient cognitive capacity to complete the questionnaire independently, and (e) providing informed consent.

The sample was nearly balanced between public and private institutions (50% each). Of the participants, 55% were women and 45% men, with a mean age of 20.44 years (SD = 2.29). Most students identified as Ecuadorian nationals (99%), single (96.7%), and without children (94%). Regarding perceived socioeconomic status, 47% classified themselves as middle class and 46.8% as upper class. No monetary incentives were provided. The average response rate across institutions was 42%.

### 2.2. Instruments

*Sociodemographic Questionnaire*. An ad hoc questionnaire was designed to gather information on age, gender, marital status, and perceived socioeconomic status.

*Psychological Capital Questionnaire (PCQ-12)*. The PCQ-12 (Martínez et al., 2019) was used to assess psychological capital through four dimensions: self-efficacy, hope, resilience, and optimism. The instrument consists of 12 items rated on a six-point Likert scale ranging from 1 (strongly disagree) to 6 (strongly agree), with higher scores indicating greater psychological capital.

In the present study, confirmatory factor analysis (CFA) supported a second-order four-factor structure with adequate fit indices, χ^2^(367) = 1732, CFI = 0.972, TLI = 0.969, RMSEA = 0.061 (90% CI [0.058, 0.063]), and SRMR = 0.041. Standardized factor loadings ranged from 0.710 to 0.883 for the first-order indicators and from 0.870 to 0.944 for the second-order construct, supporting the factorial validity of the model.

Regarding reliability and convergent validity, the dimensions demonstrated satisfactory psychometric properties. Self-efficacy showed α = 0.869, CR = 0.890, and AVE = 0.730; hope showed α = 0.888, CR = 0.903, and AVE = 0.700; resilience demonstrated α = 0.774, CR = 0.817, and AVE = 0.599; and optimism showed α = 0.840, CR = 0.865, and AVE = 0.762. All values exceeded the recommended thresholds for internal consistency (α and CR ≥ 0.70) and convergent validity (AVE ≥ 0.50).

*Perceived Stress Scale–Negative Stress Dimension (PSS-10)*. The Perceived Stress Scale (PSS-10), developed by Sheldon Cohen and colleagues (1983), adapted for Ecuador by Ruisoto et al. (2020), was used to assess perceived stress. The instrument comprises 10 items rated on a five-point Likert scale ranging from 0 (never) to 4 (very often). Following the theoretical framework of the present study, only the negative stress dimension was retained because it reflects perceived distress, emotional overload, and lack of control. This dimension includes six items (1, 2, 3, 6, 9, and 10).

CFA supported the adequacy of the negative stress dimension, showing acceptable fit indices (CFI = 0.955, TLI = 0.940, SRMR = 0.060). Standardized factor loadings ranged from 0.736 to 0.863, all statistically significant.

The negative stress dimension demonstrated satisfactory reliability and convergent validity, including Cronbach’s alpha (α = 0.891), composite reliability (CR = 0.914), and average variance extracted (AVE = 0.640).

*Patient Health Questionnaire (PHQ-4).* The PHQ-4 developed by Kurt Kroenke and colleagues (2009) was used to assess symptoms of anxiety and depression. The instrument consists of four items rated on a four-point Likert scale ranging from 0 (not at all) to 3 (nearly every day), with higher scores indicating greater psychological distress.

In the present study, CFA supported a bifactorial structure composed of anxiety and depression dimensions with adequate fit indices, χ^2^(367) = 1732, CFI = 0.972, TLI = 0.969, RMSEA = 0.061 (90% CI [0.058, 0.063]), and SRMR = 0.041. Standardized factor loadings ranged from 0.796 to 0.894, all statistically significant, supporting the factorial validity of the construct.

Regarding reliability and convergent validity, the anxiety–depression construct demonstrated satisfactory psychometric properties, including Cronbach’s alpha (α = 0.879), composite reliability (CR = 0.919), and average variance extracted (AVE = 0.741).

*Acceptance and Action Questionnaire–II (AAQ-II).* The AAQ-II, developed by Bond and colleagues (2011), was used to assess psychological inflexibility. The instrument consists of seven items rated on a seven-point Likert scale ranging from 1 (never true) to 7 (always true), with higher scores indicating greater psychological inflexibility and experiential avoidance.

In the present study, CFA supported the adequacy of the unidimensional structure, showing satisfactory fit indices, χ^2^(14) = 1030.006, CFI = 0.975, TLI = 0.962, RMSEA = 0.205, and SRMR = 0.039. Standardized factor loadings ranged from 0.781 to 0.886, all statistically significant. Given the small degrees of freedom and the ordinal nature of the data, greater emphasis was placed on CFI, TLI, and SRMR, which are considered more robust indicators under these conditions.

The AAQ-II demonstrated excellent reliability and convergent validity, including Cronbach’s alpha (α = 0.936), composite reliability (CR = 0.950), and average variance extracted (AVE = 0.731).

### 2.3. Procedure

This was a cross-sectional, non-experimental study. Data collection was conducted over a five-month period in 2020. Participants were contacted via institutional email lists and mobile messaging platforms, where they received a link to an online survey hosted on Google Forms.

The survey began with an introductory section describing the study’s objectives and including an informed consent form. Only those who explicitly provided consent were granted access to the questionnaires. The average completion time was approximately 12 min. Responses were automatically recorded by the platform and exported to Microsoft Excel for statistical analysis.

The study adhered to the ethical principles of the Declaration of Helsinki (World Medical Association, 2013). Participation was voluntary, anonymous, and confidential, and participants could withdraw at any time without repercussions.

### 2.4. Data Analysis

All statistical analyses were conducted using JASP (Jeffreys’s Amazing Statistics Program; version 0.19). Descriptive statistics (mean, minimum, maximum, standard deviation, skewness, and kurtosis) were computed for all variables. Distributional properties were evaluated through skewness and kurtosis indices. All variables showed acceptable distributional characteristics (|skewness| < 2; |kurtosis| < 7; Finney & DiStefano, 2006), supporting the use of robust structural equation modeling procedures appropriate for ordinal data.

Cases with incomplete data (<5%) were excluded via listwise deletion, under the assumption that data were missing completely at random (MCAR).

Prior to testing the structural model, confirmatory factor analyses (CFA) were conducted for all instruments to evaluate the adequacy of the measurement models and factorial validity. Internal consistency and convergent validity were assessed using Cronbach’s alpha (α), Composite Reliability (CR), and Average Variance Extracted (AVE). Values of α and CR ≥ 0.70 were considered indicative of adequate reliability, whereas AVE values ≥ 0.50 indicated satisfactory convergent validity (Hair et al., 2019). Detailed CFA results, reliability indices, and convergent validity estimates for each instrument are reported in the Section 2.2.

The structural equation model (SEM) was estimated using the Weighted Least Squares Mean and Variance adjusted estimator (WLSMV), which is recommended for ordinal Likert-type data and non-normal distributions. Given the low proportion of missing data (<5%), cases with incomplete responses were excluded using listwise deletion prior to model estimation. The WLSMV estimator provides robust parameter estimates and standard errors under conditions of non-normality and ordinal measurement (Li, 2016).

The proposed model specified Psychological Capital (PsyCap) as a higher-order latent construct, composed of self-efficacy, hope, resilience, and optimism, consistent with prior theoretical and empirical research (Luthans et al., 2007b; Luthans et al., 2015). In contrast to simpler modeling approaches, perceived stress was operationalized as a latent variable reflecting the negative stress dimension of the PSS-10, indicated by six items (PSS1, PSS2, PSS3, PSS6, PSS9, and PSS10), capturing perceived distress and lack of control.

Furthermore, anxiety–depression and psychological inflexibility were modeled as latent constructs, each represented by their respective observed indicators. This specification allows for a more accurate estimation of measurement error and improves the validity of structural relationships.

The structural model tested a mediational framework, in which PsyCap predicts negative stress, which in turn predicts anxiety–depression and psychological inflexibility. Direct paths from PsyCap to both outcomes were also included to evaluate partial mediation. Indirect effects were interpreted based on standardized path coefficients and statistical significance levels estimated within the SEM framework.

Model fit was evaluated using the chi-square to degrees of freedom ratio (χ^2^/df), the Comparative Fit Index (CFI), the Tucker–Lewis Index (TLI), the Root Mean Square Error of Approximation (RMSEA), and the Standardized Root Mean Square Residual (SRMR), following the guidelines of Hu and Bentler (1999) and Byrne (2016). Acceptable fit was defined as χ^2^/df ≤ 3, CFI and TLI ≥ 0.90, RMSEA and SRMR ≤ 0.08; excellent fit as χ^2^/df ≤ 2, CFI and TLI ≥ 0.95, RMSEA and SRMR ≤ 0.05.

## 3. Results

### 3.1. Descriptive Analysis

Table 1 presents the descriptive statistics for the study variables and their respective dimensions, including measures of central tendency (mean and median), dispersion (standard deviation and coefficient of variation), and distributional characteristics (skewness and kurtosis).

Regarding overall PsyCap, the mean score was 53.68 (SD = 13.81) out of a maximum of 72 points, indicating a moderately high level of this psychological resource among university students. The coefficient of variation (CV = 25.73%) suggests moderate dispersion and relative homogeneity in PsyCap levels within the sample.

The four PsyCap dimensions demonstrated favorable levels. To account for the different number of items comprising each subscale (hope = 4 items, self-efficacy = 3, resilience = 3, optimism = 2), mean scores were interpreted relative to their item count. Based on this adjustment, self-efficacy showed the highest average level (M/item = 4.62), followed by optimism (M/item = 4.59) and hope (M/item = 4.53), while resilience presented the lowest relative score (M/item = 4.17). Coefficients of variation ranged from 27% to 31%, indicating moderate dispersion. All dimensions exhibited negative skewness, suggesting a concentration of scores toward higher levels of PsyCap. Additionally, kurtosis values (ranging from 0.606 to 2.229) indicate moderately leptokurtic distributions, reflecting clustering around the mean with limited extreme values. These patterns support the presence of relatively stable and well-developed psychological resources in the sample.

In terms of psychological distress indicators, psychological inflexibility showed a mean score of 24.55 (SD = 11.08; max = 49), reflecting moderate levels. The relatively high coefficient of variation (CV = 45.14%) indicates substantial heterogeneity across participants. The distribution was approximately symmetric (skewness = 0.216) and platykurtic (kurtosis = −0.859), suggesting a relatively dispersed distribution of scores.

Regarding negative perceived stress (PSS-10 negative dimension), the mean score was 12.24 (SD = 5.32; max = 24), indicating a moderate level of stress associated with perceived lack of control and emotional overload. The coefficient of variation (CV = 43.5%) suggests considerable variability in stress levels across participants, which is particularly relevant for the proposed mediation model. The distribution was approximately symmetric (skewness = −0.140) and slightly platykurtic (kurtosis = −0.298), indicating acceptable distributional properties for the application of robust structural equation modeling procedures appropriate for ordinal and non-normally distributed data.

Finally, symptoms of anxiety and depression showed a mean of 4.27 (SD = 3.09) on a scale from 0 to 12, indicating generally low levels of emotional symptomatology. However, the coefficient of variation was high (CV = 72.47%), reflecting substantial interindividual variability. The distribution was positively skewed (skewness = 0.472), suggesting that most participants reported low symptom levels, although a subgroup exhibited moderate to high symptomatology. Kurtosis was slightly positive (0.387), indicating a near-normal distribution with some extreme values.

Taken together, these findings support the assumptions underlying the mediation model, as the negative perceived stress dimension exhibits sufficient variability and an approximately normal distribution, making it a suitable mediator between PsyCap and psychological distress outcomes. Moreover, the coexistence of relatively high PsyCap and heterogeneous levels of stress and distress suggests differential vulnerability patterns within the student population, reinforcing the relevance of examining indirect (mediated) effects.

### 3.2. Structural Model

**Figure 1:**
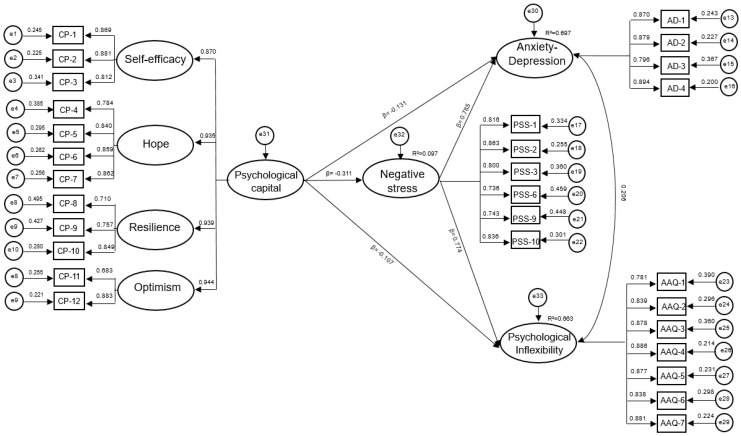
Structural equation model depicting the mediating role of negative stress in the relationship between Psychological Capital and psychological distress outcomes (anxiety–depression and psychological inflexibility).

The SEM analysis supported both the measurement and structural components of the proposed mediational model. The overall model demonstrated a good fit to the data: χ^2^(367) = 1732, *p* < 0.001; CFI = 0.972; TLI = 0.969; SRMR = 0.041; RMSEA = 0.061 (90% CI [0.058, 0.063]). Although the chi-square statistic was significant, this result was expected given the large sample size. Therefore, greater emphasis was placed on incremental and residual-based fit indices, all of which indicated an acceptable to excellent model fit. Figure 1 presents the final structural equation model with standardized parameter estimates.

Regarding the measurement model, all latent constructs exhibited strong psychometric properties. Standardized factor loadings were high and statistically significant across all indicators (λ ranging from 0.710 to 0.883 for Psychological Capital dimensions, 0.736 to 0.863 for negative stress, 0.796 to 0.894 for anxiety–depression, and 0.781 to 0.886 for psychological inflexibility; all *p*s < 0.001), supporting adequate indicator reliability. Moreover, the second-order Psychological Capital construct was strongly represented by its four dimensions, with standardized loadings ranging from 0.870 to 0.944.

Convergent validity was confirmed through the Average Variance Extracted (AVE), with all constructs exceeding the recommended threshold of 0.50. Specifically, AVE values were 0.730 for self-efficacy, 0.700 for hope, 0.599 for resilience, and 0.762 for optimism. Additionally, the AVE values for the remaining constructs were also satisfactory: 0.640 for negative stress, 0.731 for psychological inflexibility, and 0.741 for anxiety–depression.

Turning to the structural model, Psychological Capital showed a significant negative effect on negative stress (β = −0.311, *p* < 0.001), indicating that higher levels of psychological resources were associated with lower perceived stress. In turn, negative stress had strong positive effects on both anxiety–depression (β = 0.785, *p* < 0.001) and psychological inflexibility (β = 0.774, *p* < 0.001), highlighting its central mediating role within the model.

The mediation results are presented in Table 2. Negative stress significantly mediated the relationship between Psychological Capital and both psychological inflexibility and anxiety–depression. Specifically, Psychological Capital exerted significant indirect effects on psychological inflexibility (β = −0.241, *p* < 0.001) and anxiety–depression (β = −0.244, *p* < 0.001) through negative stress. Direct effects from Psychological Capital to psychological inflexibility (β = −0.107, *p* < 0.001) and anxiety–depression (β = −0.131, *p* < 0.001) remained statistically significant, indicating a pattern of partial mediation. Notably, the indirect effects were substantially larger than the direct effects, suggesting that negative stress constitutes the primary mechanism through which Psychological Capital influences psychological distress outcomes.

Furthermore, the total effects were also significant for psychological inflexibility (β = −0.348, *p* < 0.001) and anxiety–depression (β = −0.375, *p* < 0.001), reinforcing the protective role of Psychological Capital. These findings support the theoretical relevance of stress-reduction processes in explaining the association between Psychological Capital and mental health outcomes.

The model explained 9.7% of the variance in negative stress, 69.7% of the variance in anxiety–depression, and 66.3% of the variance in psychological inflexibility. These findings suggest that although Psychological Capital exerts a modest direct influence on stress, negative stress itself plays a dominant role in explaining downstream psychological distress outcomes.

Finally, a significant positive covariance was observed between anxiety–depression and psychological inflexibility (r = 0.206, *p* < 0.001), indicating that both constructs are related yet empirically distinguishable. This finding supports their conceptualization as interconnected but distinct dimensions of psychological distress.

## 4. Discussion

The present study examined the relationships among Psychological Capital (PsyCap), negative stress, anxiety–depression symptoms, and psychological inflexibility among Ecuadorian university students within an integrated transdiagnostic framework grounded in positive psychology and stress appraisal theory. Overall, the findings supported a well-fitting structural equation model in which PsyCap showed significant negative associations with negative stress and psychological distress outcomes, both directly and indirectly through negative stress. These results reinforce the relevance of PsyCap as a protective psychological resource that contributes to adaptive functioning and emotional adjustment in university populations exposed to persistent academic and psychosocial demands (Finch et al., 2025; Moreno-Montero et al., 2024; Prasath et al., 2021).

Descriptive analyses revealed moderately high levels of PsyCap among participants. Considering the different number of items across PsyCap dimensions, self-efficacy showed the highest relative score, followed by optimism and hope, whereas resilience presented comparatively lower levels. These findings suggest that students generally perceive themselves as capable of managing academic and personal demands, maintaining positive expectations regarding future outcomes, and sustaining goal-directed motivation despite adversity (Luthans et al., 2015; Youssef-Morgan & Luthans, 2015; King et al., 2020). However, the comparatively lower levels of resilience may indicate greater difficulties adapting to setbacks, uncertainty, and prolonged stress exposure, highlighting resilience as a potentially vulnerable domain among university students. This pattern is particularly relevant in demanding educational contexts characterized by socioeconomic instability and limited psychological support systems.

Regarding psychological distress, students reported moderate levels of stress and relatively lower levels of emotional symptoms, although substantial variability was observed across participants. Importantly, the present study operationalized stress specifically as negative stress, conceptualized as maladaptive stress characterized by uncontrollability, emotional overload, and dysfunctional cognitive appraisal. This distinction is theoretically meaningful because it avoids conflating stress with perceived coping capacity and provides a more precise representation of maladaptive stress experiences associated with psychological dysfunction. Likewise, mid-range levels of psychological inflexibility suggest that while some students experience rigid cognitive–emotional responses, others may benefit from greater psychological flexibility and adaptive coping capacities. These findings are consistent with previous evidence in Latin American university populations reporting elevated stress exposure alongside heterogeneous emotional adjustment profiles (Moreta-Herrera et al., 2021; Torres et al., 2017).

From a transdiagnostic perspective, the strong interrelationships observed among negative stress, anxiety–depression symptoms, and psychological inflexibility support the existence of shared vulnerability mechanisms underlying emotional maladjustment (Bond et al., 2011; Ruisoto et al., 2022; Chuquitarco Marín & López-Guerra, 2025). In particular, the SEM results demonstrated that negative stress functioned as a central mechanism linking PsyCap with emotional symptoms and psychological inflexibility. These findings align with recent Ecuadorian evidence showing that perceived stress and psychological inflexibility jointly explain substantial variance in anxiety and depression symptoms among university students (Vaca-Gallegos et al., 2025). The present study extends this literature by positioning PsyCap as a protective antecedent resource capable of buffering the impact of maladaptive stress appraisal processes.

Consistent with Hypothesis 1, PsyCap showed a significant negative association with negative stress. This finding aligns with Lazarus and Folkman’s (1984) transactional model of stress and coping, which proposes that stress responses emerge from individuals’ cognitive appraisal of environmental demands relative to available coping resources. Students with higher levels of PsyCap may therefore perceive academic and personal challenges as more manageable and controllable, reducing maladaptive stress responses characterized by helplessness, emotional overload, and uncontrollability. This interpretation is also coherent with Snyder’s (2002) hope theory, according to which individuals with stronger motivational and cognitive resources are more capable of sustaining goal-directed behavior under adverse conditions.

The findings are consistent with previous studies reporting inverse associations between PsyCap and stress-related maladjustment. Sun et al. (2022) found that PsyCap was negatively associated with psychological distress among nursing students, whereas Asheghi et al. (2020) observed that PsyCap buffered the negative effects of job stress on burnout and mental health among healthcare professionals. Similarly, Yu et al. (2025) reported that psychological capital was associated with lower burnout and more adaptive coping under stressful conditions in athletes. However, unlike prior studies that conceptualized PsyCap primarily as a mediator or moderator, the present study positioned PsyCap as an antecedent psychological resource influencing maladaptive stress appraisal processes. This distinction represents an important conceptual contribution because it suggests that PsyCap may shape how stressful experiences are cognitively interpreted before emotional symptoms fully emerge.

One of the most innovative aspects of the present study lies in the operationalization of stress as negative stress. Most previous investigations have relied on global perceived stress measures without differentiating adaptive and maladaptive stress experiences. By focusing specifically on negative stress, the present findings provide a more refined understanding of the stress processes most strongly associated with emotional maladjustment in university students. This distinction may be particularly relevant in Latin American contexts, where socioeconomic instability, academic uncertainty, and limited access to mental health services may intensify perceptions of uncontrollability and emotional overload (Gómez-Restrepo et al., 2025).

Consistent with Hypothesis 2, negative stress was positively associated with anxiety–depression symptoms and psychological inflexibility. These findings reinforce transdiagnostic perspectives suggesting that maladaptive stress appraisal processes function as shared vulnerability mechanisms underlying multiple forms of psychological distress. Previous studies have consistently linked stress with anxiety, depression, and emotional maladjustment among university students (Pascoe et al., 2020; Ruisoto et al., 2020). In Ecuadorian populations, Vaca-Gallegos et al. (2025) identified perceived stress and psychological inflexibility as central predictors of anxiety and depression symptoms. The present study extends this evidence by demonstrating that negative stress simultaneously predicts emotional symptoms and psychological inflexibility within the same structural model.

The relationship between negative stress and psychological inflexibility is particularly relevant because it suggests that maladaptive stress responses may reinforce rigid patterns of experiential avoidance and cognitive fusion. According to Acceptance and Commitment Therapy models, psychological inflexibility limits individuals’ capacity to respond adaptively to internal experiences and environmental demands (Bond et al., 2011). Students experiencing elevated negative stress may therefore become increasingly vulnerable to avoidance-based coping strategies, emotional dysregulation, and persistent anxiety–depression symptoms. In this sense, stress may represent not only an emotional reaction to academic demands, but also a broader cognitive–emotional vulnerability process that interferes with adaptive functioning.

In line with Hypothesis 3, PsyCap demonstrated significant direct negative effects on anxiety–depression symptoms and psychological inflexibility. These findings indicate that PsyCap contributes to psychological adjustment not only through stress reduction processes but also through direct pathways associated with emotional regulation and adaptive functioning. Previous research has shown that PsyCap is positively associated with psychological well-being, academic engagement, resilience, and adaptive coping strategies (Datu & Valdez, 2016; Finch et al., 2020; Moreno-Montero et al., 2024). Moreno-Montero et al. (2024), for example, found that PsyCap positively predicted psychological well-being and adaptive coping while negatively predicting maladaptive coping strategies among university students. The present findings extend this literature by demonstrating that PsyCap is also inversely associated with psychological inflexibility, suggesting that positive psychological resources may facilitate greater emotional openness, behavioral flexibility, and adaptive regulation of internal experiences.

Importantly, the stronger association observed between PsyCap and negative stress compared with direct emotional outcomes suggests that positive psychological resources may primarily operate through cognitive appraisal processes. Students with higher levels of hope, self-efficacy, optimism, and resilience may reinterpret stressful situations more adaptively, thereby reducing the escalation of emotional symptoms. This interpretation strengthens the integration between positive psychology and stress appraisal theory by suggesting that PsyCap shapes how adversity is cognitively processed before emotional distress intensifies.

Consistent with Hypothesis 4, negative stress significantly mediated the relationship between PsyCap and both anxiety–depression symptoms and psychological inflexibility. The SEM results indicated partial mediation, as both direct and indirect effects remained statistically significant. These findings suggest that PsyCap contributes to psychological adjustment not only directly, but also indirectly through reductions in maladaptive stress appraisal processes. Students with higher PsyCap may perceive stressful situations as less threatening and more controllable, thereby reducing dysfunctional cognitive–emotional responses associated with anxiety, depression, and psychological rigidity.

These mediation findings extend previous literature in conceptual and methodological terms. Whereas prior studies primarily examined PsyCap as a mediator or moderator of stress-related outcomes (Asheghi et al., 2020; Sun et al., 2022; Wang et al., 2021), the present study tested a model in which PsyCap functions as an antecedent factor influencing downstream psychological outcomes through negative stress. Furthermore, unlike previous investigations focused on isolated indicators of maladjustment, the current study simultaneously evaluated anxiety–depression symptoms and psychological inflexibility within a single structural equation model. This integrated approach provides a broader understanding of the shared mechanisms underlying emotional dysfunction in university students and strengthens the transdiagnostic contribution of the study.

Regarding Hypothesis 5, the indirect effects through negative stress were stronger than the direct effects, supporting a partial mediation model. These findings suggest that one of the principal mechanisms through which PsyCap contributes to mental health is by reducing maladaptive stress experiences. In practical terms, PsyCap may enhance students’ capacity to reinterpret stressful situations more adaptively, thereby reducing perceptions of helplessness, overload, and emotional exhaustion that intensify psychological distress.

The structural model explained a substantial proportion of variance in anxiety–depression symptoms and psychological inflexibility, indicating strong explanatory capacity. Specifically, approximately 67% of the variance in anxiety–depression symptoms and 65% of the variance in psychological inflexibility were explained by the proposed model. These findings highlight the value of integrating positive psychology, stress appraisal theory, and transdiagnostic perspectives within a unified explanatory framework. Moreover, modeling PsyCap as a second-order latent construct composed of self-efficacy, hope, resilience, and optimism further supports its theoretical validity as a multidimensional psychological resource relevant for academic contexts (Martínez et al., 2019; Luthans et al., 2007b).

### 4.1. Theoretical Implications

From a theoretical standpoint, the findings demonstrate that PsyCap operates not only as a psychological strength associated with well-being, but also as a cognitive–emotional resource capable of shaping maladaptive stress appraisal processes. The study also extends Lazarus and Folkman’s (1984) stress appraisal framework by empirically demonstrating that positive psychological resources may influence emotional outcomes indirectly through reductions in maladaptive stress responses. Furthermore, conceptualizing negative stress as a transdiagnostic mechanism advances previous research that relied primarily on global perceived stress measures, offering a more nuanced understanding of the mechanisms underlying psychological vulnerability among university students.

### 4.2. Practical Implications

The findings have important practical implications for higher education institutions, particularly in Latin American contexts where university students are frequently exposed to academic pressure, socioeconomic instability, and limited access to mental health services. The results suggest that interventions should not only strengthen PsyCap dimensions (such as hope, resilience, self-efficacy, and optimism) but also directly target maladaptive stress appraisal processes.

Universities may benefit from implementing evidence-based psychoeducational programs focused on adaptive coping, emotional regulation, resilience training, and stress management strategies (Luthans et al., 2007b; Youssef-Morgan & Luthans, 2015). Interventions aimed at strengthening PsyCap could include goal-setting workshops to enhance hope (Luthans et al., 2015), self-regulated learning strategies to improve self-efficacy (King et al., 2020), adaptive coping practices to strengthen resilience (Coutu, 2002), and cognitive restructuring or positive reappraisal techniques to foster optimism (Seligman, 2002).

Importantly, the present findings indicate that these interventions should be integrated with approaches specifically designed to reduce negative stress, including mindfulness-based interventions, stress inoculation training, Acceptance and Commitment Therapy strategies, and cognitive-behavioral techniques aimed at modifying maladaptive stress appraisal patterns. Embedding these combined interventions within university counseling and student support services may be particularly relevant in contexts where stress, anxiety, and depressive symptoms are highly prevalent (Moreta-Herrera et al., 2021; Vaca-Gallegos et al., 2025).

The findings further suggest that strengthening PsyCap and reducing maladaptive stress perceptions may contribute not only to improved mental health but also to better academic engagement, persistence, resilience, and overall well-being, consistent with prior research in Latin American and international contexts (Datu & Valdez, 2016; Ortega-Maldonado & Salanova, 2017; Moreno-Montero et al., 2024). In resource-limited settings, PsyCap-based interventions may represent a cost-effective and scalable preventive strategy because they can be delivered through brief structured programs, online platforms, and group-based psychoeducational approaches.

### 4.3. Limitations and Future Directions

Several limitations should be acknowledged. Firstly, the cross-sectional design prevents establishing temporal precedence or causal relationships among PsyCap, negative stress, anxiety–depression symptoms, and psychological inflexibility. Although the proposed structural model was theoretically grounded, reciprocal or bidirectional relationships among these variables remain plausible. Longitudinal and experimental studies are therefore necessary to clarify causal pathways and examine the temporal stability of these associations (Finch et al., 2025; Moreno-Montero et al., 2024).

Secondly, all variables were assessed using self-report instruments, which may increase the risk of common method variance, shared response bias, and social desirability effects (Brenner & DeLamater, 2016). Consequently, some associations may have been inflated by shared subjective variance rather than reflecting exclusively substantive psychological processes. Future studies should incorporate multi-method assessment strategies, including behavioral indicators, ecological momentary assessment, physiological stress markers, academic performance indicators, or informant-based reports to strengthen methodological robustness (Rosenman et al., 2011).

Thirdly, although all major study variables were modeled as latent constructs, some differences remained in the complexity of the measurement models across constructs. Specifically, Psychological Capital was specified as a second-order latent construct, whereas anxiety–depression, negative stress, and psychological inflexibility were modeled as first-order latent variables. Although this approach allowed adequate control of measurement error, future studies may benefit from testing alternative measurement structures and longitudinal latent models to further strengthen parameter estimation and construct representation.

Another limitation concerns the contextual specificity of the sample. Participants were exclusively university students from Loja, Ecuador, recruited through non-probabilistic procedures. Therefore, the findings should be interpreted cautiously and may not generalize to students from other regions or sociocultural contexts. Future studies should examine whether the proposed transdiagnostic model operates differently across public and private universities, academic disciplines, socioeconomic groups, and culturally diverse populations, including rural, Indigenous, and socioeconomically vulnerable students.

Although conceptualizing stress as negative stress represents an innovative contribution, additional psychometric and longitudinal evidence is required to determine its discriminant validity relative to general perceived stress, emotional dysregulation, and related constructs. Finally, the present model did not include other potentially relevant psychological and contextual variables, such as coping strategies, emotional regulation, social support, academic burnout, sleep quality, loneliness, spirituality, and family functioning, which may influence the relationship between PsyCap and psychological distress (Khan et al., 2024). Future studies should therefore explore more comprehensive models incorporating additional mediating and moderating mechanisms to better understand the pathways through which PsyCap influences mental health outcomes.

Overall, the present study contributes to the growing literature on PsyCap and student mental health by proposing an integrated transdiagnostic framework in which PsyCap functions as an antecedent psychological resource influencing emotional adjustment through maladaptive stress appraisal processes. By introducing the construct of negative stress and simultaneously examining anxiety–depression symptoms and psychological inflexibility within a single structural equation model, the study advances previous research and provides new evidence regarding psychological vulnerability and adaptive functioning among Ecuadorian university students.

## 5. Conclusions

The present study provides robust empirical evidence supporting the protective role of Psychological Capital (PsyCap) in relation to psychological distress among Ecuadorian university students. The findings demonstrated that PsyCap was negatively associated with negative stress, anxiety–depression symptoms, and psychological inflexibility, both directly and indirectly through maladaptive stress appraisal processes. These results reinforce the relevance of PsyCap as a key psychological resource that promotes adaptive functioning and emotional adjustment in demanding academic contexts.

One of the principal contributions of the study lies in conceptualizing negative stress as a central transdiagnostic mechanism linking PsyCap with emotional maladjustment. Unlike previous research focused primarily on global perceived stress, the present findings suggest that maladaptive stress responses characterized by uncontrollability, emotional overload, and dysfunctional cognitive appraisal play a particularly important role in explaining psychological vulnerability among university students. In this sense, the study advances the current literature by demonstrating that PsyCap may influence mental health not only directly, but also indirectly through the cognitive and emotional interpretation of stressful experiences.

The findings further reinforce the value of transdiagnostic perspectives for understanding university student mental health. The significant associations observed among negative stress, anxiety–depression symptoms, and psychological inflexibility support the existence of interconnected psychological processes underlying emotional vulnerability. Moreover, the study extends previous literature by positioning PsyCap as an antecedent psychological resource capable of shaping stress appraisal processes, rather than conceptualizing it solely as a mediator or moderator within stress-related models.

From a theoretical perspective, the findings strengthen the integration between positive psychology and Lazarus and Folkman’s (1984) stress appraisal framework by demonstrating that positive psychological capacities may reduce emotional distress through their influence on maladaptive stress responses. Consequently, the proposed model offers an integrative framework for understanding how psychological strengths, stress appraisal processes, and emotional vulnerability interact within university populations.

From a practical standpoint, the findings highlight the importance of implementing university-based interventions aimed at strengthening resilience, hope, optimism, and self-efficacy while simultaneously reducing maladaptive stress appraisal patterns. Interventions grounded in positive psychology, emotional regulation, adaptive coping, and stress management strategies may help students respond more effectively to academic and psychosocial demands, thereby reducing vulnerability to anxiety, depression, and psychological inflexibility.

Overall, the study contributes to the growing literature on PsyCap and student mental health by proposing an innovative transdiagnostic framework tested within an underrepresented Latin American context. In increasingly demanding academic environments, strengthening psychological capacities such as hope, resilience, optimism, and self-efficacy may represent a relevant pathway for promoting psychological well-being and reducing stress-related vulnerability among university students.

Finally, future longitudinal, experimental, and cross-cultural studies are needed to clarify causal relationships and further examine the mechanisms through which PsyCap influences mental health over time. Future research should also explore additional psychological and contextual variables that may shape the relationship between positive psychological resources, maladaptive stress processes, and emotional adjustment in university populations.

## Figures and Tables

**Table 1:** Descriptive statistics of the study variables and their dimensions.

Study Variables	M	Me	SD	CV	AS	K	Min	Max
Psychological Capital	53.68	57	13.81	25.73%	−1.385	2.229	0	72
Self-efficacy	13.85	15	3.99	28.81%	−1.395	1.786	0	18
Hope	18.14	19	5.00	27.56%	−1.366	1.961	0	24
Resilience	12.51	13	3.839	30.69%	−0.864	0.606	0	18
Optimism	9.18	10	2,658	28.95%	−1.239	1.444	0	12
Psychological Inflexibility	24.55	24	11.081	45.14%	0.216	−0.859	7	49
Perceived stress (negative dimension)	12.24	12	5.320	43.5%	−0.140	−0.298	0	24
Anxiety/Depression	4.27	4	3.094	72.47%	0.472	0.387	0	12

Note: M = mean; Me = median; SD = standard deviation; CV = coefficient of variation; AS = skewness; K = kurtosis; Min = minimum; Max = maximum. PsyCap subscales include different item numbers (hope = 4, self-efficacy = 3, resilience = 3, optimism = 2), with corresponding maximum scores of 24, 18, 18, and 12. For interpretation, mean scores were also considered relative to the number of items in each subscale.

**Table 2:** Direct, indirect, and total effects of Psychological Capital on psychological distress outcomes through negative stress.

Outcome	Effect Type	β (Std.)	SE	z	*p*	95% CI
Psychological inflexibility	Direct (c_1_)	−0.107	0.015	−7.11	<0.001	[−0.137, 0.078]
	Indirect (a × b_1_)	−0.241	0.018	−13.57	<0.001	[−0.276, 0.206]
	Total	−0.348	0.021	−16.25	<0.001	[−0.390, −0.306]
Anxiety–depression	Direct (c_2_)	−0.131	0.018	−7.31	<0.001	[−0.166, 0.096]
	Indirect (a × b_2_)	−0.244	0.018	−13.49	<0.001	[−0.280, 0.209]
	Total	−0.375	0.023	−16.50	<0.001	[−0.420, 0.330]

Note. β = standardized coefficient; SE = standard error; CI = confidence interval. Negative stress significantly mediated the relationship between Psychological Capital and both psychological inflexibility and anxiety–depression. The indirect effects were substantially larger than the direct effects, supporting a partial mediation model.

## Data Availability

The data presented in this study are available on reasonable request from the corresponding author. Restrictions apply due to confidentiality agreements and institutional regulations. The author will provide anonymized datasets to qualified researchers for purposes of verification, replication, or further academic inquiry.

## Abbreviations

The following abbreviations are used in this manuscript:

AAQ-II	Acceptance and Action Questionnaire-II
AVE	Average Variance Extracted
CFA	Confirmatory Factor Analysis
CFI	Comparative Fit Index
CI	Confidence Interval
CR	Composite Reliability
CV	Coefficient of Variation
EJIHPE	European Journal of Investigation in Health, Psychology and Education
GAD-2	Generalized Anxiety Disorder-2
IBM	International Business Machines
PCQ-12	Psychological Capital Questionnaire-12
PHQ-2	Patient Health Questionnaire-2
PHQ-4	Patient Health Questionnaire-4
PsyCap	Psychological Capital
PSS-10	Perceived Stress Scale-10
RMSEA	Root Mean Square Error of Approximation
SD	Standard Deviation
SEM	Structural Equation Modeling
SRMR	Standardized Root Mean Square Residual
TLI	Tucker–Lewis Index
UTPL	Universidad Técnica Particular de Loja
WLSMV	Weighted Least Squares Mean and Variance Adjusted
